# Pomegranate peel methanolic‐extract improves the shelf‐life of edible‐oils under accelerated oxidation conditions

**DOI:** 10.1002/fsn3.1391

**Published:** 2020-03-05

**Authors:** Abdalla E. El‐Hadary, Mohamed Taha

**Affiliations:** ^1^ Department of Biochemistry Faculty of Agriculture Benha University Toukh Egypt; ^2^ Centre for Environmental Sustainability and Remediation RMIT University Bundoora Melbourne VIC Australia

**Keywords:** corn oil, HPLC, natural antioxidant, phenolic and flavonoid fractions, pomegranate peel extract, soybean oil, sunflower oil

## Abstract

Natural antioxidants extracted from agri‐waste resources have gained increased economic, sustainable, and health attention due to their sustainability, safer food‐applications, and beneficial components. Pomegranate peel extracts (*Punica Granatum* L.) have natural phytochemicals with superior protective effects stabilizing a variety of the most common vegetable oils consumed globally. Among five different pomegranate peel extracts, methanolic extract has maximum total phenolic content of 18.89%, a total flavonoid content of 13.95 mg QE kg^−1^, and a relative antioxidant activity of 93% when compared to other pomegranate peel extracts. Additionally, the HPLC analysis of pomegranate peel methanolic extract exhibited the maximum number of phenolic and flavonoid fractions. HPLC fractions showed that pyrogallol and ellagic acids were the most abundant phenolic compounds with 453 and 126 mg kg^−1^, respectively. In terms of flavonoid fractions, hesperidine and quercetrin were the highest detected‐flavonoids with about 50 and 35 mg kg^−1^, respectively, from HPLC flavonoids fractions. Therefore, pomegranate peel methanolic extract was selected at different concentrations (100, 200, 400, and 600 ppm) for the stabilizing experiment of Egyptian freshly refined edible oils (sunflower, soybean, and corn oils) in comparison with synthetic antioxidant (tert‐butyl hydroquinone TBHQ‐200 ppm) during accelerated storage at 70°C for 10 days. The results from the accelerated storage experiment indicated that pomegranate peel methanolic extract (at different concentrations: 200, 400, and 600 ppm) exhibited stronger antioxidant capability in all tested oils rather than negative controls (without antioxidant) and synthetic antioxidant TBHQ‐200. Under accelerated oxidation conditions, pomegranate peel methanolic extract have the potential capability to improve the shelf life of edible oils in comparison with the most powerful synthetic antioxidant (TBHQ‐200 ppm).

## Graphical abstract

The idea of using agricultural waste for the generation of powerful antioxidant is brilliant especially when applied on large scale. Pomegranate peel methanolic extract has the capability to improve the shelf life of edible oils under accelerated oxidation conditions when compared to synthetic antioxidants.

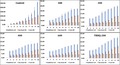

## 1 INTRODUCTION

Vegetable oils originating from vegetable resources (such as sunflower, soybean, and corn) are high in polyunsaturated fatty acids and highly recommended globally as a human cooking media that have many beneficial and nutritional effects on the human body (Derakhshan et al., 2018; Mei et al., 2014; Mohdaly, Sarhan, Mahmoud, Ramadan, & Smetanska, 2010; Mohdaly, Smetanska, Ramadan, Sarhan, & Mahmoud, 2011). Frying and deep‐frying processes are used in everyday activities such as takeaways and fast‐food which are becoming more popular than ever before. The unique sensory characteristics of fried‐food are very popular worldwide (Bopitiya & Madhujith, 2014). The chemical structure of fatty acids and their derivatives (triglycerides) are unstable especially when exposed to extreme environmental factors such as constant high temperature, air, and light. Therefore, fatty acids and their derivatives are susceptible to chemical oxidation which results in producing an unpleasant flavor and consequently shortens their shelf life. Oils undergo further oxidation (rancidity) due to chemical or environmentally improper storage conditions which modifies their organoleptic characteristics and negatively affects shelf life and their nutritional and economic characteristics (Ibrahium, 2010; Mohdaly et al., 2010). Consequently, the nutritional quality and economic value of these rancid oils is reduced significantly resulting in potential economic loss.

It has been reported that chemical deterioration (lipid peroxidation) of food products (mainly fatty/lipids products) are the main cause of food spoilage leading to consumer dissatisfaction and economic crisis (Ibrahium, 2010; Padmaja & Prasad, 2011). Oxidative stability parameters of edible oils were varied significantly from one oil to another and might be progressed from a few days to a few months ending with the formation of rancid oxidative primary and secondary products (Mohdaly et al., 2010, 2011). The formation of primary and secondary oxidative products (mixtures of volatile aldehyde compounds) requires regular analysis (aging tests) for each batch on a regular basis to evaluate the validity and estimate the shelf life of oily products (Mohdaly et al., 2010). Konsoula (2016) reported that to estimate the oxidative status of any edible oil, four different oxidation parameters such as peroxide values, conjugated dienes, conjugated trienes, and ρ‐anisidine values should be routinely monitored and estimated from each batch on a large scale (Konsoula, 2016).

Oily food products require a protective agent against auto‐oxidation and chemical spoilage during their shelf life storage (Ibrahium, 2010). Therefore, the addition of antioxidant agents in the food industry is highly required not only to preserve desirable taste, color, and flavor but also to overcome the stability problems and to increase the shelf life of oils and their derivatives (Mohdaly et al., 2010). Since the beginning of the twentieth century, synthetic antioxidants such as butylated hydroxyanisole (BHA), butylated hydroxytoluene (BHT), and tert‐butyl hydroquinone (TBHQ) have been widely used commercially as potential synthetic antioxidants mainly for oils due to their high content of polyunsaturated fatty acids (Derakhshan et al., 2018; Padmaja & Prasad, 2011). Food additives such as synthetic antioxidants have been the most applicable and effective methods to prevent oxidation, peroxidation, and auto‐oxidation of oily products. Synthetic antioxidants have the capability to stabilizing oil characteristics, preventing oil rancidity, delaying oil deterioration and increasing the shelf life of lipids and lipid‐containing products (Konsoula, 2016; Mohdaly et al., 2010, 2011; Padmaja & Prasad, 2011). Although synthetic antioxidants have significant capability in industrial practices to reduce damage and chemical spoilage caused by oxidizing agents (such as free radicals), previous researchers have confirmed the potential health risks and negative impacts of the long‐term commercial use of synthetic antioxidants (Hygreeva, Pandey, & Radhakrishna, 2014; Mohdaly et al., 2011). Synthetic antioxidants might be responsible for the formation of peroxyl and hydroxyl free radicals and other secondary toxic compounds, which might lead to serious human health concerns and might be associated with carcinogenic, mutagenic, and aging effects (Bopitiya & Madhujith, 2014; Ibrahium, 2010; Mohdaly et al., 2010, 2011; Padmaja & Prasad, 2011).

For instance, TBHQ was globally considered as the most powerful synthetic antioxidant for many years; however, it has been recently prohibited in many developed countries such as Europe, Canada, and Japan (Konsoula, 2016; Mohdaly et al., 2010). According to the US Food & Drug Administration (https://www.fda.gov/default.htm), BHA has also been removed from the Generally Recognized as Safe (GRAS) list of compounds for more than a decade (Konsoula, 2016). Therefore, the idea of overcoming undesirable side effects and replacing the addition of synthetic antioxidants with natural antioxidants (plant origin) on the large scale remains a current area of interest especially when extracted from cheap, abundant, and sustainable agricultural wastes (Bopitiya & Madhujith, 2014; Ibrahium, 2010; Konsoula, 2016). Since natural antioxidants have become more preferred than synthesized antioxidants, agricultural wastes are considered as an alternative, low‐cost source of natural antioxidants especially when extracted and applied to the oil industry commercially.

Currently, the exploration for natural antioxidants extracted from abundant, sustainable, cheap, and environmentally friendly resources (including agri‐waste) has become a popular topic for the food industry and has been more preferred globally by consumers than using toxic‐synthesised antioxidants (Basiri, 2015; Bopitiya & Madhujith, 2014; Ibrahium, 2010; Padmaja & Prasad, 2011). After confirming the negative effects of over using synthetic antioxidants (on the long‐term), the continuous application of synthetic antioxidants is becoming more restricted globally due to their toxicity and health risks (Ibrahium, 2010; Konsoula, 2016).

Food industries have faced the challenge by looking for natural, safe, economic, and effective antioxidants originated from vegetables, fruits, plants, and agricultural‐residues such as wheat and rice bran, peanut hulls, and old tea leaves (Konsoula, 2016). Vegetables, fruits, and their residues are a cheap and abundant rich‐source of natural polyphenolic and flavonoid compounds which could play an important role not only as antioxidants (by inhibiting lipid oxidation), but also as antimicrobial agents by inhibiting microbial growth and preventing several diseases (Basiri, 2015; Hygreeva et al., 2014). During fruit processing and food industry processes, fruit peel is normally generated in high amount during the season (as a low‐cost agri‐waste) and considered as a promising source for natural antioxidant. Natural antioxidants could be extracted and effectively exploited due to their dual effects: increasing the shelf life of edible oils (by preventing rancidity) and preventing microbial contamination (Bopitiya & Madhujith, 2014; Derakhshan et al., 2018; Ibrahium, 2010).

Pomegranate fruit (*Punica granatum* L. *Punicaceae*) is one of the oldest edible fruits widely cultivated in many tropical and subtropical countries. It has been cultivated in far‐East countries including India, China, Pakistan, Iran and stretching globally to the Middle‐East and European continent through to the Mediterranean region (Konsoula, 2016). In ancient cultures (such as Indian and Egyptian), this fruit was commonly known as a “Paradise fruit” due to its extensive use in folk medicine (Basiri, 2015; Derakhshan et al., 2018; Hygreeva et al., 2014). It has been reported that pomegranate peels have been used by ancient Egyptians and Indians as a therapeutic agent with medicinal properties against several ailments including cough, diarrhea, dysentery, dental plaque, inflammation, ulcers, bleeding noses, infertility, and intestinal worms (Ismail, Sestili, & Akhtar, 2012). Additionally, pomegranate peel extract has the capability to treat some chronic diseases due to its anticancer properties such as colon and prostate cancer, melanogenesis (skin cancer), breast cancer and stomach ulcers. In addition, pomegranate juice has the capability (as a powerful agent) to treat a variety of health problems such as Alzheimer's disease, asthma, prostate cancer, piles, diarrhea, stomach ache, coughing, sneezing, skin inflammation, piles, and hyperacidity (Basiri, 2015; Derakhshan et al., 2018; Hygreeva et al., 2014). Pomegranate fruit or juice is considered a good source of many vital vitamins, anti‐inflammatory compounds, natural estrogens, and essential minerals (Basiri, 2015). Furthermore, due to the excellent antioxidant activity of pomegranate peel extract, it has exhibited the potential activity as a cardiovascular protective agent inhibiting the formation and the accumulation of foam cells and cholesterol in the arteries (Basiri, 2015; Hygreeva et al., 2014).

Pomegranate fruit can be consumed fresh or in any processed forms such as fresh juice, jelly, beverages, jam, or wines (Basiri, 2015). Pomegranate fruit can also be used as a natural flavoring or coloring agent. It has been reported that pomegranate juice product has a high nutritional value due to the possession of the highest antioxidant activities when compared with commonly known antioxidant‐rich beverages such as green tea, fruit juices, and red wine (Wang, Pan, Ma, & Atungulu, 2011). According to Food and Agriculture Organization's statistic (FAO) in 2012, the global annual production of pomegranate fruit reached 1.5 million tons (Malviya, Jha, & Hettiarachchy, 2014). The processing industry of pomegranate fruits resulted in generating large quantities of agri‐waste materials (such as peels) that could cause environmental concerns when incorrectly disposed and might add extra cost for this agri‐waste to be collected, treated, and disposed of appropriately (Mohdaly et al., 2010; Padmaja & Prasad, 2011; Taha et al., 2016). It has been reported that pomegranate fruit consists of two edible parts (seeds and arils) and two nonedible parts (exocarp and mesocarp) (Bopitiya & Madhujith, 2014; Derakhshan et al., 2018; Konsoula, 2016). The interior and exterior membranes of pomegranate fruit are nominated as “Peels” derived from hull and pericarp that comprise approximately 30%–60% of the total weight (Malviya et al., 2014). Rahnemoon, Jamab, Dakheli, and Bostan (2016) reported that approximately 40% of pomegranate fruit is peel and this valuable part is normally wasted (Rahnemoon et al., 2016). Although pomegranate peels are considered an agri‐waste, the bioactive compounds are concentrated in the peels rather than in the aril, seeds, and leaves (Rahnemoon et al., 2016). Pomegranate derivatives are an important source of phenolic and flavonoid fractions that potentially contribute to the antioxidant activity. Thus, peel extract has been reported to have a relative antioxidant activity higher than in seed and pulp (Wang et al., 2011). The pomegranate peels were found to have a significantly higher content of phenolic and flavonoid compounds which are a powerful source of natural antioxidants (Konsoula, 2016). Pomegranate peel is one of the most beneficial parts when compared to the seeds, leaf, and flower due to its substantial amounts of bioactive ingredients such as polyphenolic and flavonoid compounds that can be attributed to the antioxidant index (Konsoula, 2016; Malviya et al., 2014). This might be due to the high content of polyphenolic compounds found in peel (~73%) compared to the polyphenolic compounds from seeds (~27%) (Wang et al., 2011). Pomegranate peel methanolic extract has shown the highest antioxidant activity over different ranges of polar and nonpolar extracts (Toklu et al., 2007). Besides its antioxidant properties when applied to edible oils, pomegranate peel also been shown to have antimicrobial activities, therefore playing a dual role (Al‐Zoreky, 2009; Kanatt, Chander, & Sharma, 2010; Rahnemoon et al., 2016; Rosas‐Burgos et al., 2017).

Pomegranate peel might be a safer natural supplement acting as a potential (dual) antioxidant and antimicrobial agent rather than using toxic synthetic antioxidant and antimicrobial agents (Ibrahium, 2010; Rahnemoon et al., 2016; Rosas‐Burgos et al., 2017). Pomegranate peel potentially possesses higher amounts of polyphenolic content and antibacterial and antifungal activities suggesting its dual role as natural antioxidant and antimicrobial agent (Ibrahium, 2010; Malviya et al., 2014; Rahnemoon et al., 2016; Wang et al., 2011). It has been recently reported that the high amount of tannins such as punicalagin found in pomegranate peel extracts might be a key factor responsible for its antimicrobial activity (Ibrahium, 2010; Rosas‐Burgos et al., 2017). The reason might be the high amount of polyphenols and tannins have the capability to denature microbial cellular membranes which leads to microbial death (Ibrahium, 2010; Rahnemoon et al., 2016).

Therefore, the main objective of this study was to assess and evaluate the replacement of pomegranate peel methanolic extract as a natural antioxidant (at different concentration levels: 100, 200, 400, and 600 ppm) during the accelerated oxidation conditions of sunflower, soybean, and corn oils by measuring primary and secondary oxidation parameters in comparison with the widespread synthetic antioxidant TBHQ (200 ppm). This study attempted also to identify the antioxidant phenolic and flavonoid compounds present in the methanolic extracts using high‐performance liquid chromatography (HPLC) techniques.

## 2 MATERIALS AND METHODS

### 2.1 Materials

Sunflower, soybean, and corn crude oils (free of any antioxidant) were obtained (freshly & refined) from Arma Company (https://www.arma.com.eg/; 10th of Ramadan City, Egypt). Folin–Ciocalteu's phenol reagent, 2, 2‐diphenylpicrylhydrazyl (DPPH), 2,2′‐azino‐bis (3‐ethylbenzothiazoline‐6‐sulfonic acid) diammonium salt (ABTS), synthetic tertiary butylhydroquinone (TBHQ), ρ‐Anisidine, gallic acid, quercetin, and all organic solvents were purchased from Sigma Company. Pomegranate fruit (the natural source of antioxidants) was obtained from Egyptian local Market.

### 2.2 Methods

#### 2.2.1 Preparation of pomegranate peel extracts

Ten kilograms of pomegranate fruit was purchased from Egyptian local market. For sample preparation of pomegranate peel extracts, seeds were separated manually from the peel, then the separated peels were cut into small pieces, and then allowed to air‐dry in the dark at room temperature until constant weight was achieved. Air‐dried peel was then homogenized using a coffee grinder until a fine powder was obtained. A constant amount of peel powder (20 g) was used in order to extract natural antioxidants (in triplicate) using a variety of different organic solvents such as petroleum ether, ethyl acetate, ethanol 80% (v/v), methanol 80% (v/v), and water for 15 hr using Soxhlet apparatus. All individual extracts were then combined in a brown bottle, filtered through 45 µm sterilized filters, and evaporated to dryness under vacuum using a mini‐rotary evaporator (N‐N series, EYELA) at 40°C until almost all the solvent was removed. Semi‐dried extracts of each solvent were stored at −20°C for further use (Rahnemoon et al., 2016; Toklu et al., 2007).

#### 2.2.2 Selection of oils

The selection of vegetable oils was based on variations in polyunsaturated fatty acid composition. Because sunflower oil is rich in linoleic acid, soybean oil includes a high level of α‐linolenic acid. Also, corn oil is mainly composed of polyunsaturated fatty acids that is relatively low in saturated fat and very rich in linoleic acid (Mohdaly et al., 2010).

#### 2.2.3 Determination of total phenolic content

Total phenolic content was determined according to Singleton and Rossi (1965) using Folin–Ciocalteu reagent and gallic acid (as standard) with slight modifications (Singleton & Rossi, 1965). One milligram of each individual extract was dissolved in 5 ml of distilled water (in triplicate), and 0.5 ml (in triplicate) was separated in fresh tubes for the total flavonoid content. Then, 1 ml of sodium carbonate (20%; w/v) was mixed in a tube containing 3.5 ml extract and 1 ml of Folin–Ciocalteu reagent. The mixture was gently mixed and allowed to stand in a water bath at 40°C for 30 min. The absorbance of the color developed (purple‐bluish) was measured at 765 nm using a UV‐Vis spectrophotometer (UV‐1800 spectrophotometer, TOMOS) against the extract free controls. Total phenolic content was calculated using a gallic acid standard curve.

#### 2.2.4 Determination of total flavonoid content

Total flavonoid content of all the different extracts was determined using a minor modification of the method reported by (Meda, Lamien, Romito, Millogo, & Nacoulma, 2005). One milligram of each extract was dissolved in 5 ml of distilled water, and then, 0.5 ml was transferred in fresh tubes (in triplicate), then mixed with 0.5 ml methanol (80% V/V), 50 µl potassium acetate (1 M), and 1.4 ml water, and incubated at room temperature for 30 min. The absorbance of the reaction mixture was measured at 415 nm, and the total flavonoid content was calculated using quercetin as a standard curve.

#### 2.2.5 Determination of antioxidant activity

Determination of the free radical scavenging activity was done using 2, 2‐diphenylpicrylhydrazyl (DPPH) as a substrate according to Choi et al. (2002) with minor modifications. Substrate‐methanol stock solution (0.004% w/v) was freshly prepared for all samples, controls, and standard curve. One mg of each sample was dissolved in 100 µl methanol solution (including negative controls); then, 3.9 ml of methanol stock solution (0.004% w/v) was added to all samples for dilution. Vigorous shaking was done at room temperature for 15 min, and then, all samples were allowed to stand in the dark for 30 min for the color development. Absorbance of all samples (including samples, blank, controls, and standard) was measured at 517 nm using UV‐Vis spectrophotometer. Ascorbic acid was used in different concentrations for blotting the standard curve. The percentage of DPPH was calculated according to this equation % DPPH = [100 × (A1–A2)/A2]; where A1 is control absorbance, and A2 is sample absorbance (pomegranate peel extracts) (Choi et al., 2002).

#### 2.2.6 HPLC analysis

The semi‐dried methanolic extract was initially dissolved in HPLC grade methanol (1.0 mg/ml), then filtered through a sterile Millipore filter (0.22 µm), and subjected to qualitative and quantitative analysis by HPLC (Shimadzu LC‐IOA). The instrument was equipped with a dual‐pump LC‐1 OAT binary system with a UV detector SPD‐10A and a Phenomenex Luna RP, C18 column (4.6 s 250 mm). Data were integrated by Shimadzu Class VP series software. Separation was achieved using aqueous acetonitrile as a mobile phase containing 1% acetic acid. The linear gradient program started with 18% acetonitrile, changing to 32% in 15 min and finally to 50% in 40 min. Results were obtained by comparison of peak areas (λmax = 254 nm) of the samples (mg/g dry extract) (Prakash, Singh, & Upadhyay, 2007).

#### 2.2.7 Comparison of different levels of natural antioxidants against TBHQ 200 ppm

Natural antioxidant (methanolic extract) was added as pomegranate peel extract (PPE) to sunflower, soybean, and corn oils (individually) at four concentration levels (100, 200, 400, and 600 ppm) in comparison with both negative controls (without antioxidant) and positive controls using synthetic antioxidant (TBHQ 200 ppm) as recommended by previous studies (Mohdaly et al., 2010). Negative controls were conducted under the same conditions for each individual oil (in triplicate) without the addition of any synthetic antioxidant or pomegranate peel extract. All treatments were conducted in triplicate and run in opened transparent jars (500 ml) which enabled direct interaction between oil samples, light, surface, and atmospheric air. These jars were kept in an oven at 70°C for 10 days (Konsoula, 2016). Synthetic antioxidant (TBHQ) and pomegranate peel methanolic extract were dissolved in propylene glycol prior to the addition of all oil treatments. An aliquot (30 ml) was collected from each treatment periodically every 0, 2, 4, 6, 8, and 10 days for the measurement of peroxide values (PV), conjugated dienes (CD), and conjugated trienes (CT) and determination of secondary oxidation products [ρ‐anisidine values (ρ‐AV)].

#### 2.2.8 Determination of primary oxidation products

Peroxide values (PV) from all treatments were measured using iodometric titration methods (Shahidi & Wanasundara, 2002). A known weight approximately 2.0–5.0 g of oil sample was weighed into a 250 ml Erlenmeyer dried flask, and then, 10 and 15 ml of chloroform and glacial acetic acid were added, respectively. The flasks were shaken until the oil was completely dissolved, and then, one ml of saturated potassium iodine solution (KI) was added. The flasks were then closed and left to stand for 5 min in the dark. After that, 30 ml of distilled water was added. The titration was slowly conducted using a standard solution of sodium thiosulfate (0.01 N) in the presence (few drops) of starch indicator.

#### 2.2.9 Conjugated dienes and conjugated trienes

Specific extinctions (presented in units; U) at λ 233 nm and 268 nm were determined for the conjugated dienes and conjugated trienes, respectively, using a spectrophotometer (UV‐1800 spectrophotometer, TOMOS, Italy). Oil samples were diluted with isooctane following the standard method of IUPAC method II. D.23 (IUPAC, 1979). The absorbance (U) measured at λ 233 and 268 nm was referred to the conjugated diene (CD) and conjugated triene (CT) of polyunsaturated fatty acids (PUFAs), respectively. Increasing absorption (U) values (at λ 233 nm and 268 nm) were considered as an indication of proceeding oil oxidation. From the results obtained, the concentration of conjugated diene [CD] was calculated as follows:

CD=A/l×ε.

CD refers to the conjugated diene value.

1. where A = Absorbance at λ 233 nm.
2. l = Optical path of the cuvette 1 cm.
3. ε = 2.525 × 104 M − 1·cm − 1 = Molar absorptivity of Linoleic Acid Hydroperoxide.

CD value=CD×2.5×104/m.

where: 2.5 × 104 is the correction factor; m, mass of oil sample.

#### 2.2.10 Determination of secondary oxidation products [ρ‐anisidine value (ρ‐AV)]

ρ‐AV was determined according to the AOCS Official Method Cd18‐90 (AOCS, 1995). The oil sample (0.5–2.0 g) was diluted to 25 ml with isooctane in a volumetric flask. The oil solution (5 ml) was mixed with 1 ml of 0.25% p‐anisidine in glacial acetic acid, and after 10 min, the absorbance at 350 nm was read using a spectrophotometer (UV‐1800 spectrophotometer, TOMOS, Italy) against a reagent blank. The p‐AV is given by the formula: ρ‐AV = 25 × (1.2As–Ab)/m where As = Absorbance of the oil solution after reaction with the p‐anisidine reagent, Ab = Absorbance of the oil solution alone, and m = Mass of the sample (g).

### 2.3 Statistical analyses

Statistical analyses were conducted using SPSS (version 16.0). All treatments and analyses were performed in triplicate, and all mean values, standard deviations, and standard errors were calculated using Microsoft Excel software.

## 3 RESULTS AND DISCUSSION

### 3.1 Chemical composition of pomegranate peel powder

Traditional chemical composition was conducted on pomegranate peel fine powder and the approximate percentage of crude carbohydrates (78%), fiber (12%), protein (3.5%), ash (3.4%), and lipids (2.25%) were measured and calculated on a dry weight basis shown in Table 1. The chemical composition of pomegranate peel powder was mainly lignocellulosic carbohydrates which were mainly structural carbohydrates, cellulose, hemicellulose, and lignin.

**Table 1:** Chemical composition of pomegranate peel powder (% on dry weight basis)

Components	Pomegranate peel (%)
Moisture	12.15 (±0.52)
Ether extract	2.20 (±0.08)
Crude protein	3.55 (±0.06)
Crude ash	3.43 (±0.04)
Crude fiber	12.04 (±0.06)
Crud carbohydrate	77.64 (±0.36)

### 3.2 Total phenolic, flavonoid, and antioxidant activity of different pomegranate peel extracts

*Punica granatum* L. is known as “pomegranate” or “Paradise fruit” in many ancient cultures, and this fruit and its derivatives (especially peels) are rich in beneficial secondary metabolites (phytochemicals) and have potential economic, nutritional, and medicinal benefits globally due to their wide range of unique properties such as antioxidant, antibacterial, anti‐atherosclerotic, anti‐allergic, and anti‐inflammatory characteristics (Elfalleh et al., 2012; Khalil, Khan, & Shabbir, 2018; Padmaja & Prasad, 2011). The extraction efficiency of their phytochemical components (secondary metabolites) depends on some key factors such as the pomegranate part (leaves, peels, seeds, and flowers), pomegranate cultivars, solvent types, sample**:** solvent ratio, extraction duration, pressure, and extraction temperature (Ardekani et al., 2011). Among all the pomegranate parts, pomegranate peels have shown the maximum polyphenolic and flavonoid content followed by flowers, leaves, and seeds (Elfalleh et al., 2012). Konsoula (2016) found that pomegranate peel extracts contained polyphenolics approximately three‐ to fivefold higher than the existence of seed and juice extracts, respectively (Konsoula, 2016). That is why pomegranate peel was selected in this study and phytochemical components were extracted using a variety of different solvents. The polarity of extracting solvents was also known to play a critical role in the extraction process and might change the quantity and quality of the extracted (macro or micro) molecules. To the author's knowledge, there is no powerful solvent or combination of different solvents (in specific ratio) that has the maximum extraction capability to extract the diverse range of chemical fractions from pomegranate peel powder (Singh et al., 2014).

Herein, five different solvents with different polarity strength were used to compare the extraction efficiency of natural molecules extracted from pomegranate peel powder (Table 2). Under the same extraction conditions, the number of extracted fractions, total phenolic content, total flavonoid content, and antioxidant activity are significantly different from one solvent to another. In this study, the methanolic extract obviously has more fractions (35) than all of the ether (2), ethyl acetate (1), ethanol (9), and water (10) (Table 2). Approximately, 41% of the extracted phenolic content was found in the methanolic extract and around 20% was found in the ethanolic extract. The same trend but more pronounced was clearly observed in the extraction of the total flavonoid molecules; methanolic extract alone has the ability to extract more than 52% of the total flavonoid contents, and the rest of the four solvents were able to extract about 48% of the total flavonoids content (Table 2). Consequently, the antioxidant activity of the methanolic extract was the highest (94%) among ether (12%), ethyl acetate (26%), ethanol (74%), and water (72%). In a comparative study aimed to extract total polyphenolic and flavonoid components from pomegranate leaves, peels, and seeds using water and methanol, pomegranate peel methanolic extract had higher polyphenolic (86%) and flavonoid (52%) content than water extract (54% and 21%, respectively) followed by pomegranate leaves methanolic extract and pomegranate seed methanolic extract (Elfalleh et al., 2012). A current study conducted by Konsoula (2016) who extracted bioactive compounds from pomegranate peel, seed, and juice using methanol and ethanol solvents found that pomegranate peel methanolic extract had better antioxidant index than pomegranate peel ethanolic extract. Pomegranate peel methanolic extract had the highest yield (29%) when compared to seed (20%) and juice (14%). In addition, pomegranate peel methanolic extract had the highest polyphenolic content (190 mg/g) compared to seeds (70 mg/g) and juice (34 mg/g) and the highest amount of flavonoid (21 mg/g), compared to seeds (9 mg/g) and juice (4 mg/g) (Konsoula, 2016). That is why pomegranate peel was chosen herein for extracting the maximum phytochemical phenolic and flavonoid components.

**Table 2:** Total phenolic content, total flavonoid content, and antioxidant activity of different pomegranate peel extracts

Pomegranate peel	% Total of extracts/fractions	%Total phenolic content	Total flavonoid content (mg QE/kg extract)	%Antioxidant activity
Ether extract	2.25 ± 0.09	3.11 ± 0.003	0.39 ± 0.13	11.97 ± 0.64
Ethyl acetate extract	1.00 ± 0.09	6.50 ± 1.85	1.86 ± 0.29	26.15 ± 1.17
Ethanol extract	9.05 ± 0.34	9.00 ± 0.08	3.44 ± 0.25	74.27 ± 0.49
Methanol extract	**35.40 ± 1.44**	**18.89 ± 0.11**	**13.95 ± 0.95**	**93.97 ± 1.91**
Water extract	10.62 ± 0.23	8.11 ± 0.56	7.4 ± 0.06	72.27 ± 0.76

Bold indicates the methanolic extract as the main significant result.

Therefore, our findings were supported by previous results where methanol is the most preferable solvent for extraction of the majority of polyphenolic and flavonoid compounds from pomegranate samples (Elfalleh et al., 2012; Konsoula, 2016; Padmaja & Prasad, 2011). The efficiencies of the solvents used for extraction of the antioxidant phenolics were in the order: methanol > ethanol>water > ethyl acetate > ether. The reason for this order might be due to the solubility of phenolic and flavonoid compounds which are affected by the polarity of solvents. For instance, polyphenolic compounds are polar and easily get dissolved in polar solvents such as aqueous methanol and faced difficulties with nonpolar solvents such as ether (Basiri, 2015; Khalil et al., 2018; Konsoula, 2016). That is why nonpolar solvents such as ether, ethyl acetate, and chloroform always showed the lowest extracted phytochemical fractions (Padmaja & Prasad, 2011). These results are in agreement with the findings confirmed by previous researchers (Basiri, 2015; Elfalleh et al., 2012; Khalil et al., 2018; Padmaja & Prasad, 2011; Singh, Chidambara Murthy, & Jayaprakasha, 2002).

Although previous study showed that hexane and acetone had the highest extraction efficiencies for pomegranate defatted seed, methanolic seed extract showed the highest phenolic content (28 mg/L) when extracted with hexane (0.29 mg/L), ethyl acetate (0.37 mg/L), butanol (0.57 mg/L), acetone (3.41 mg/L), and water (22.61 mg/L) (Basiri, 2015). Therefore, the effective extraction of polyphenolic compounds was normally achieved by methanolic or aqueous‐methanolic extract (Basiri, 2015; Derakhshan et al., 2018; Elfalleh et al., 2012; Khalil et al., 2018; Padmaja & Prasad, 2011; Singh et al., 2002). Pomegranate peel methanolic extract showed the most significant free radical scavenging capability rather than other tested extracts and this is in agreement with previous literature. Therefore, pomegranate peel methanolic extract was selected for HPLC characterization and further investigations.

### 3.3 Characterization of pomegranate peel methanolic extract using HPLC

Pomegranate peel methanolic extract was further characterized by HPLC for the presence of the two main phytochemical compounds; polyphenolic and flavonoid fractions and all detected fractions were illustrated in (Table 3). Generally, twenty‐two polyphenolic fractions and twenty flavonoid compounds were separately detected in the fractionation of pomegranate peel methanolic extract at different retention times. In terms of phenolic compounds, pyrogallol, ellagic acid, ρ‐hydroxy‐benzoic acid, catechol, and catechin were the highest detected phenolic compounds with approximately 454, 126, 70, 60, and 33 (mg kg^−1^ dry weight), respectively (Table 3). Apart from this, hesperidine and quercetrin were the highest flavonoid compounds observed in the pomegranate peel methanolic extract with about 50 and 35 (mg kg ^−1^ dry weight), respectively (Table 3). In general, the total phenolic content in pomegranate seed methanolic extract is relatively high with approximately 894 mg/g (the sum of all phenolic fractions in Table 3) and almost the same as 867 mg/g determined in a different study (Ibrahium, 2010). The concentration of ellagic acid was sevenfold (126 mg/g) the amount found in pomegranate peel ethanolic extract (18 mg/g) (Ibrahium, 2010). These qualitative analyses of the total phenolic compounds are in agreement with Madrigal‐Carballo, Rodriguez, Krueger, Dreher, & Reed, (2009) who found that the number of total phenolics varied in pomegranate cultivars (19 different types) and ranged between 354 and 783 mg/g (Madrigal‐Carballo et al., 2009).

**Table 3:** Phenolic compounds and flavonoid compounds of pomegranate peel extract (methanolic extract) analyzed by HPLC

Phenolic compounds	Phenolic fractions (mg kg^−1^)	Flavonoids compounds	Flavonoids fractions (mg kg^−1^)
Gallic	25.00	Apig.6‐arbinose 8‐galactose	3.53
Pyrogallol	453.58	Apig.6‐rhamnose 8‐ galactose	7.19
4‐Amino‐benzoic	0.84	Naringin	9.44
Protocatchuic	19.87	Luteo.7‐glucose	6.22
Catechein	32.75	Rutin	2.65
Chlorogenic	15.62	Hesperidine	50.47
Catechol	59.65	Quercetrin‐3‐O‐glucose	1.89
Caffeine	13.39	Kamp.3,7‐dirhamoside	3.23
P‐OH‐benzoic	70.17	Apig.7‐O‐neohesperidoside	3.29
Caffeic	4.58	Quercetrin	35.19
Vanillic	8.05	Apigenin‐7‐glucose	7.8
p‐coumaric	0.86	Kaemp.3‐(2‐p‐coumaroyl) glucose	10.24
Ferulic	4.92	Quercetin	2.15
Iso‐ferulic	1.17	Acacetin7 neo hesperside	3.42
Rosmarinic	11.73	Naringenin	0.89
Ellagic	125.61	Hespirtin	5.23
Benzoic	6.66	Acacetin neo.rutinoside	1.21
Alpha‐coumaric	2.68	Rhamentin	3.51
3,4,5‐methoxy‐cinnamic	1.64	Apegnin	1.07
Coumarin	9.12	Kaempferol	1.06
Salicylic	1.08		
Cinnamic	25.00		

The previous study conducted on pomegranate aril showed that ellagic acid was detected in different extracts except for the water extract, and it was the highest detected phenolic compound with 34.5 (μg/mg) when a combination of ethanol: ether: water (8:1:1) was used (Singh et al., 2014). Bopitiya and Madhujith (2014) concluded that the presence of significant amounts of phenolic and flavonoid compounds (such as ellagic and gallic acids) were attributed to the antioxidant properties of pomegranate peel extracts (Bopitiya & Madhujith, 2014).

Examination of the scientific literature regarding the correlation between the total polyphenolic profile from pomegranate peel methanolic extract and the antioxidant index found the composition of polyphenolic fractions varied significantly (depends on many factors) and the combination/ratio of polyphenolics worked in a synergy to retard oil deterioration and each phenolic fraction exhibited different thermal stability (Konsoula, 2016; Madrigal‐Carballo et al., 2009; Singh et al., 2014). All of the detected phytochemical components (functional compounds) were factors in the antioxidant activity of pomegranate peel methanolic extract, and this confirms its capability to improve the shelf life of edible oils (sunflower, soybean, and corn oils) rather than using commercially synthetic antioxidant (TBHQ‐200 ppm) under accelerated oxidation conditions.

### 3.4 Effect of methanolic extract on sunflower, soybean, and corn oils under accelerated oxidation conditions

Six different treatments were conducted on three different edible oils under accelerated oxidation conditions (at 70°C for 10 days) in order to evaluate the oxidation defence activity of pomegranate peel methanolic extract at different concentration levels (100, 200, 400, and 600 ppm) in comparison with negative control (without any antioxidant) and positive control (using synthetic TBHQ‐200 ppm) (Ibrahium, 2010; Mohdaly et al., 2010). The primary (i.e., peroxide value) and secondary (i.e., anisidine value) oxidative products were measured daily (routinely), and each concentration level of pomegranate peel methanolic extract was compared and assessed individually against positive and negative controls.

### 3.5 Changes in peroxide values of sunflower, soybean, and corn oils during accelerated oxidation conditions

**Figure 1:**
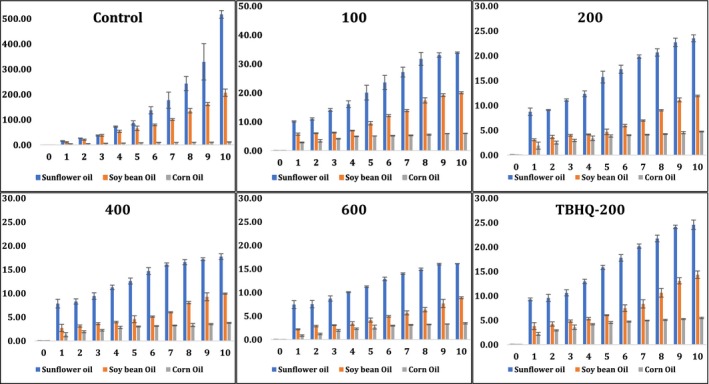
Changes in peroxide values (meq/kg) of sunflower, soybean, and corn oils during accelerated oxidation conditions using pomegranate peel methanolic extract at different concentrations (100, 200, 400, and 600 ppm) in comparison with negative control (without any antioxidant) and positive control (using synthetic TBHQ‐200 ppm)

Peroxide value is a common measure for the formation of primary oxidative products (rancidity) such as peroxides and hydro‐peroxides in the initial stages of oil oxidation (Mohdaly et al., 2010). Initial increase rate in peroxide values was very slow except the negative control treatments (with no antioxidants) which started to increase faster after 5 days reaching 87, 66, and 8 meq/kg in sunflower, soybean, and corn oil, respectively (Figure 1). A significant difference (*p* < .05) in peroxide values were observed between the negative control and all treatments containing pomegranate peel methanolic extracts and synthetic antioxidants. Under accelerated oxidation conditions (during 10 days), the effect of different antioxidants (pomegranate peel methanolic extract at 100, 200, 400, 600, and TBHQ‐200 ppm) was significant in reducing peroxide values in all oils when compared to the negative control. For instance, at the end of the accelerated oxidation experiment (after 10 days), the peroxide value was developed rapidly and reached 517, 207, and 12 meq/kg in the (negative) controls of sunflower, soybean, and corn oils, respectively (Figure 1). The high amount of peroxide values in the negative controls indicated the greatest intensity of primary oxidative products. The peroxide values were significantly (*p* < .05) reduced to 34, 20, and 6 meq/kg in sunflower, soybean, and corn oils, respectively, when pomegranate peel methanolic extract was used at 100 ppm. These numbers were significantly (*p* < .05) reduced more to 24, 12, and 5 meq/kg in sunflower, soybean, and corn oils, respectively, when pomegranate peel methanolic extract was used at 200 ppm. The peroxide values were 25, 14, and 5 meq/kg in sunflower, soybean, and corn oils, respectively, when synthetic antioxidant TBHQ‐200 was used which is very close to the value obtained when pomegranate peel methanolic extract 200 ppm was used. Interestingly, there was no significant difference between peroxide values when pomegranate peel methanolic extract was used at 400 and 600 ppm but both treatments have the significant capability (*p* < .05) to reduce the peroxide values more than synthetic antioxidant TBHQ‐200 in all tested oils (Figure 1). These numbers reduced to 18, 10, and 4 meq/kg and to 16, 9, and 3 meq/kg in sunflower, soybean, and corn oils when pomegranate peel methanolic extract was used at 400 and 600 ppm, respectively (Figure 1). Generally, pomegranate peel methanolic extracts (400 and 600 ppm) were more superior than TBHQ‐200.

In another study conducted by Konsoula (2016), pomegranate peel methanolic extract (1,000 ppm) had the capability to reduce the peroxide value of corn oil after 10 days of accelerated oxidation conditions from 346 meq/kg to 65 meq/kg, while synthetic antioxidant (BHT‐200 ppm) was only able to reduce the peroxide value to approximately 80 meq/kg (Konsoula, 2016). Additionally, it was clear that pomegranate peel methanolic extract (1,000 ppm) was more effective in preventing the formation of primary oxidation products in corn oil than pomegranate seed and juice methanolic extracts (Konsoula, 2016).

**Figure 2:**
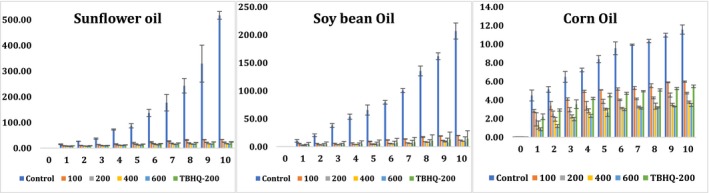
Changes in peroxide values of each individual oil (sunflower, soybean, and corn oils) during accelerated oxidation conditions using pomegranate peel methanolic extract at different concentrations (100, 200, 400, and 600 ppm), negative control (without any antioxidant) and positive control (TBHQ‐200 ppm)

On the other hand, peroxide values were significantly (*p* < .05) differed from one oil to another after 10 days accelerated oxidation. The maximum peroxide value was recorded in the negative control of sunflower oil with about 517 meq/kg at day 10 of accelerated oxidation conditions, while corn oil was the lowest in peroxide value (at the same time and conditions) with only about 12 meq/kg (Figure 2). Therefore, sunflower oil samples seem to develop their peroxide values more rapidly than corn oil in their negative controls when no antioxidants were added. It has been reported that peroxide values of the sunflower oil were consistently higher than corn oil at various stages of aging (Huang, Hsieh, Huang, & Chang, 1981). As a result, pomegranate peel methanolic extract was highly recommended at both levels (400 and 600 ppm) rather than using synthetic antioxidant (TBHQ‐200) for a wide variety of edible oils.

Generally, antioxidants (natural or synthetic) are proposed not only to delay the auto‐oxidation of oils but also to delay the accumulation of primary and secondary oxidation products, and therefore, the oxidative stability and the shelf life of these oils improved significantly (*p* < .05). To confirm the results of peroxide values of the three individual edible oils with all different treatments, other oxidation parameters (i.e., conjugated dienes, conjugated trienes, and ρ‐anisidine values) should be monitored (Mohdaly et al., 2010).

### 3.6 Changes in conjugated dienes of sunflower, soybean, and corn oils during accelerated oxidation conditions

**Figure 3:**
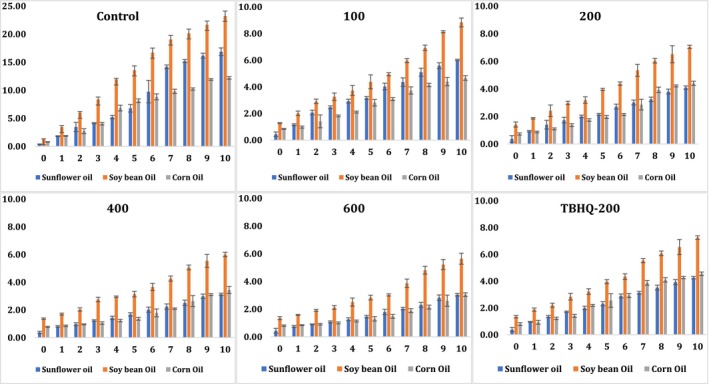
Changes in conjugated diene (U; λ_233 nm_) of sunflower, soybean, and corn oils during accelerated oxidation conditions using pomegranate peel methanolic extract at different concentrations (100, 200, 400, and 600 ppm) in comparison with negative control (without any antioxidant) and positive control (using synthetic TBHQ‐200 ppm)

Changes in the content of conjugated dienes were a good indicator to follow lipid oxidation during accelerated oxidation conditions against edible oils (sunflower, soybean, and corn) in the presence and absence of natural antioxidant extract or synthetic antioxidant (Ibrahium, 2010; Konsoula, 2016). It has been reported that the production of the high content of conjugated dienes might be related to the high content of polyunsaturated fatty acids in edible oils (Konsoula, 2016). The relative increase in the conjugated dienes was used as a parameter for the measurement of oxidative deterioration of oils and indicates the effectiveness of the best treatment that could be exploited as natural antioxidants in a variety of different edible oils. Rather than sunflower oil which has the highest peroxide values (in all conducted treatments), soybean oil had the highest content of conjugated dienes and corn oil had the lowest (Figure 3). It might be due to the high linolenic acid content in soybean oil which undergoes auto‐oxidation (Huang et al., 1981). Highest content of conjugated dienes was observed for controls (without any antioxidants), indicating higher intensity of oxidation under the accelerated storage conditions, followed by pomegranate peel methanolic extract (100 ppm), synthetic antioxidant (TBHQ‐200 ppm), and pomegranate peel methanolic extract at levels of 200, 400, and 600 ppm increasing the stability of all edible oils. Without the addition of any antioxidants (control), soybean was the highest at day 10 (with about 23 U) followed by sunflower (with approximately 17 U) and corn oil with the lowest (12 U). All the treatments of supplementing edible oils with pomegranate peel methanolic extract, except at a concentration of 100 ppm decreased more in the content of conjugated dienes than synthetic TBHQ‐200. The addition of pomegranate peel methanolic extract at a level of 400 ppm and the synthetic TBHQ‐200 resulted in a significant decrease (*p* < .05) in the conjugated dienes content in all oils when compared to controls. For instance, the conjugated dienes were 3.13, 6.0, and 3.44 U when pomegranate peel methanolic extract (400 ppm) was added to sunflower, soybean, and corn oil, respectively. However, when TBHQ‐200 was added, the content of conjugated dienes was 4.26, 7.25, and 5.55 U in sunflower, soybean, and corn oil, respectively (Figure 3). In terms of shelf life and increasing stability of oils, it can be concluded that pomegranate peel methanolic extract (at level 200, 400, and 600 ppm) achieved improvements over supplementing all edible oils with synthetic TBHQ‐200. As a result, pomegranate peel methanolic extract was highly recommended at levels (200, 400, and 600 ppm) rather than using synthetic antioxidant (TBHQ‐200) for a wide variety of edible oils.

### 3.7 Changes in conjugated trienes of sunflower, soybean, and corn oils during accelerated oxidation conditions

**Figure 4:**
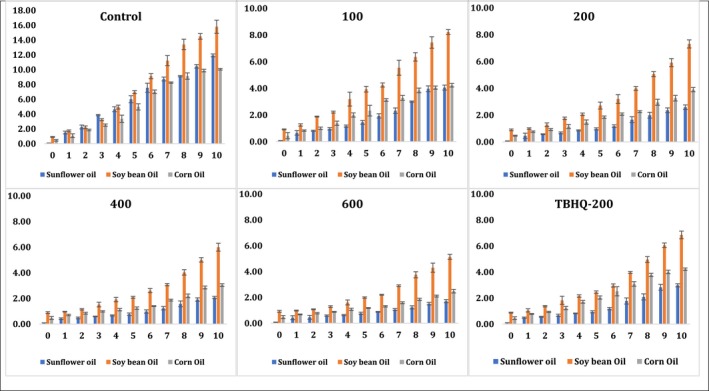
Changes in conjugated triene (U; λ_268 nm_) of sunflower, soybean, and corn oils during accelerated oxidation conditions using pomegranate peel methanolic extract at different concentrations (100, 200, 400, and 600 ppm) in comparison with negative control (without any antioxidant) and positive control (using synthetic TBHQ‐200 ppm)

The content of conjugated trienes was used as a good indicator to investigate the relationship between the oxidation of edible oils (sunflower, soybean, and corn) and the supplementation of pomegranate peel methanolic extract and synthetic antioxidant (TBHQ‐200) as protective agents (Ibrahium, 2010; Konsoula, 2016). The relative increase of the content of conjugated dienes and trienes was proportional to the interaction between edible oils and oxygen (Konsoula, 2016). It has been reported that the production of a high content of conjugated trienes might be related to the high content of dehydration of conjugated diene hydroperoxide products (Konsoula, 2016). Initial increase was observed in the content of conjugated trienes; over the time studied, values started to increase faster daily. Not much difference was observed in the content of conjugated dienes and trienes. Soybean oil in all treatments (including negative control) achieved a higher content of conjugated trienes than sunflower and corn oils and corn oil had the lowest rate increase over the accelerated oxidation period. Some concentrations of pomegranate peel methanolic extract (especially 400 and 600 ppm) had the capability to protect edible oils from the formation of primary and secondary oxidation products during the accelerated oxidation experiment compared to the addition of applying synthetic antioxidant (TBHQ‐200) (Figure 4). Without any addition of natural extract or synthetic antioxidants during the accelerated oxidation experiment, negative control treatments reached the highest in the conjugated trienes content in all tested oils (Figure 4). By the end of the accelerated oxidation experiment, the content of conjugated trienes reached about 12, 16, and 10 U in sunflower, soybean, and corn oils, respectively (Figure 4). Then, the addition of synthetic antioxidant (TBHQ‐200) reduced these values to approximately 3, 7, and 4 U in sunflower, soybean, and corn oils, respectively (Figure 4). No significant differences (*p* < .05) were observed between applying synthetic antioxidant and pomegranate peel methanolic extract (at levels 200 and 400 ppm) (Figure 4). However, the best significant result (*p* < .05) was observed when pomegranate peel methanolic extract (600 ppm) was applied (in all oils) as the conjugated trienes content was reduced to 1.7, 5, and 2.5 U in sunflower, soybean, and corn oils, respectively (Figure 4). Therefore, pomegranate peel methanolic extract at different levels (200 and 400 ppm) might have the same antioxidant activity as TBHQ or much better at higher concentration (600 ppm) and could be exploited as a safer, cheaper, and environmentally friendly solution to the oil industry (Figure 4).

### 3.8 Changes in ρ‐anisidine value of sunflower, soybean, and corn oils during accelerated oxidation conditions

**Figure 5:**
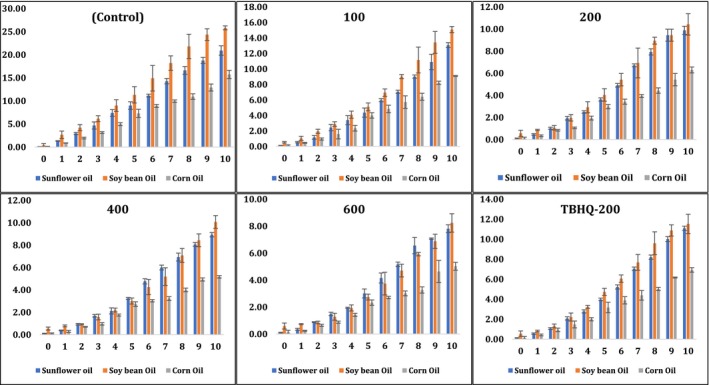
Changes in p‐anisidine value (p‐AV) of sunflower, soybean, and corn oils during accelerated oxidation conditions using pomegranate peel methanolic extract at different concentrations (100, 200, 400, and 600 ppm) in comparison with negative control (without any antioxidant) and positive control (using synthetic TBHQ‐200 ppm)

Primary and secondary oxidation products were monitored during an accelerated oxidation experiment of three edible oils in order to evaluate the significant differences between the addition of pomegranate peel methanolic extract (at different levels) and applying TBHQ‐200 (Figure 5). ρ‐anisidine is a well‐known colorimetric reaction used to measure the presence of secondary oxidation products in oils. At termination of the accelerated oxidation storage, ρ‐anisidine results were in good agreement with the primary oxidative product parameters such as peroxide value, conjugated diene, and conjugated triene content (Figure 5). As the accelerated oxidation time increased, all the parameters monitored for the primary and secondary oxidation products increased. The increased rate might be slightly different for different oil types and the type of treatment. Soybean oil in all treatments (including negative controls) had the highest ρ‐anisidine value followed by sunflower oil and corn oil. As corn oil showed the lowest increase in the primary oxidation parameters (peroxide value, content of conjugated dienes and trienes) over the storage time, the formation of secondary oxidation products (ρ‐anisidine) was also the lowest for corn oil (Figure 5). Negative controls exhibited the highest content of ρ‐anisidine followed by the addition of pomegranate peel methanolic extract at a level of 100 ppm, TBHQ‐200, then pomegranate peel methanolic extract at levels of 200, 400, and 600 ppm. Therefore, there was no significant difference between the addition of pomegranate peel methanolic extract (200 ppm) and the synthetic antioxidant (200 ppm). However, the addition of pomegranate peel methanolic extract (600 ppm) showed a significant difference in the formation of ρ‐anisidine when compared to TBHQ‐200 (Figure 5).

Previous work conducted on applying pomegranate peel ethanolic extract at two levels (400 and 800 ppm) against the oxidation of sunflower oil in comparison with synthetic antioxidant (BHT 200 ppm) showed an enhanced oxidative stability of sunflower oil at a level of 800 ppm (Ibrahium, 2010). In another study, pomegranate peel extract appeared to be effective in delaying corn oil deterioration when applied at 1,000 ppm (Konsoula, 2016). However, the oxidative stability of pomegranate peel extracts varied dramatically between extracts and our recorded results herein exhibited a good antioxidant capacity at the lower levels of 200, 400, and 600 ppm due to the high levels of phenolic compounds extracted by the most effective scavenger solvent (methanol) among other extracts. Furthermore, Konsoula (2016) noticed that the methanolic extract of pomegranate peel, seed, juice (1,000 ppm), and synthetic antioxidant (BHT‐200 ppm) has the capability to reduce the ρ‐anisidine value of corn oil by 5.3‐, 3.3‐, and 4.3‐fold, respectively, when compared to controls (without any antioxidants) (Konsoula, 2016). Therefore, pomegranate peel methanolic extract exhibited higher antioxidant activity than pomegranate seed, juice extracts (1,000 ppm), and synthetic antioxidant (BHT‐200 ppm) (Konsoula, 2016). Our finding to some extend at our tested concentrations of pomegranate peel methanolic extract (200, 400, and 600 ppm) were much promising than previous studies under the same conditions (Ibrahium, 2010; Konsoula, 2016).

## 4 CONCLUSION

Plant extracts could play an important role as an alternative to synthetic antioxidants in the oil industry. Pomegranate peel is considered a cheap, abundant, enriched source of functional components and a sustainable source for the extraction of polyphenolic and flavonoid compounds which have a remarkable antioxidant capacity. Among different pomegranate peel extracts, pomegranate peel methanolic extract had the highest of the total extracted fractions, total phenolic content, and total flavonoid content and was the highest in antioxidant activity. All the monitored (primary and secondary) oxidative products on the three edible oils were stabilized and were significantly lower than the negative controls and synthetic antioxidant (TBHQ‐200) at the termination of the accelerated oxidation storage. All edible oils supplemented with pomegranate peel methanolic extract and synthetic antioxidants (TBHQ‐200) showed low peroxide values, conjugated diene, conjugated triene content, and ρ‐anisidine value when compared with negative controls (without any antioxidants). This indicated that the pomegranate peel methanolic extract at levels 200, 400, and 600 ppm showed a good antioxidant activity so that there was no need to apply synthetic antioxidant. Our results also confirmed that the replacement of synthetic antioxidant with pomegranate peel methanolic extract could lead to an increase in thermal resistance, oxidative stability, and shelf life storage of three edible oils individually at lower doses (200, 400, and 600 ppm) when compared to the literature.

## CONFLICT OF INTEREST

The authors declare that they have no conflict of interest.

## ETHICAL STATEMENT

Human and animal testing is unnecessary in this study. Therefore, this article does not contain any studies with human participants, animal studies, clinical trial, and biosecurity that requiring ethical approval.
